# MED10/372: Access to Current Information in Africa: How easy is it?

**DOI:** 10.2196/jmir.1.suppl1.e53

**Published:** 1999-09-19

**Authors:** O Johnson

**Affiliations:** ^1^Africa Telehealth Project, OttawaCanada

**Keywords:** Medical Education

## Abstract

**Introduction:**

Health professionals need access to current information and world literature to carry out their tasks effectively. This involves quick and reliable access to world health literature, learning modules, discussions with colleagues world-wide. It also required skills and the ability to store and retrieve information. Because of the increasing costs burden, the libraries in industrially developing countries, and indeed in the developed countries, are unable to maintain subscriptions to core medical journals and books. Even in ideal situations, physical and timely access to printed resources is difficult. The use of information communication technology (ICT) offers the possibility of access to information at any time and from anywhere. There are still many barriers, such as lack of infrastructure and the cost of access, but these will be surmounted to make information accessible from the fingertips.

**Methods:**

Development of local resources. Access to local information, health statistics, available human and physical resources, and reliable communication with peers and colleagues can be greatly enhanced by use of information technology. Information resources that address local needs and are maintained by local people are important. Simple communications can go a long way to save time, exchange ideas and energy and bring rapid relief to those in needs. It can also reduce much waste. Up-to-date health statistics on disease and treatment plans can make it possible to make informed and timely decisions by health professionals. Discussion groups amongst health professionals can bring the synergy and exchange of valuable advice and resources in local communities.

**Results:**

We have identified the need of:

- Sustainability of these resources.
- Development and support of local expertise.

**Discussion:**

Africa has a population of approximately 800 million people. Of this, approximately 65% live in the rural areas. Many of these people live in isolated villages with poor road access. Their access to health care is limited to rural clinics and hospitals, often more than a days walk from their residence. Once they have reached these facilities, there is often no way of transferring them to large institutions since the cost of such transport is often has to be borne by the patient and is outside the means of most in the rural areas. Few emergency transport services exist in the rural areas. As a result, doctors in Africa are forced into carrying out most procedures themselves, often with little training and no specialist back up. While this means that African rural practitioners are highly skilled in themselves, the need for specialist back is apparent. This will also serve to decrease the isolation that these

